# Contemporary Role of Cardiac Magnetic Resonance in the Management of Patients with Suspected or Known Coronary Artery Disease

**DOI:** 10.3390/medicina57070649

**Published:** 2021-06-24

**Authors:** George Bazoukis, Stamatis S. Papadatos, Archontoula Michelongona, Konstantinos Lampropoulos, Dimitrios Farmakis, Vassilis Vassiliou

**Affiliations:** 1 Department of Cardiology, Larnaca General Hospital, 6051 Larnaca, Cyprus; 2 Department of Anatomy, Histology and Embryology, School of Health Sciences, University of Ioannina, 451 10 Ioannina, Greece; stamspap@gmail.com; 3 Cardiology Department, “Tzaneio” General Hospital of Piraeus, 185 36 Piraeus, Greece; pas.mixelogona@hotmail.com; 4 Department of Cardiology, General Hospital of Athens “Evangelismos”, 106 76 Athens, Greece; konlampropoulos@yahoo.gr; 5 Department of Pathophysiology, School of Medicine, European University of Cyprus, 1678 Nicosia, Cyprus; 6 Shakolas Educational Center for Clinical Medicine, University of Cyprus Medical School, Palaios Dromos Lefkosias Lemesou No.215/6, Aglantzia, 2029 Nicosia, Cyprus; farmakis.dimitrios@ucy.ac.cy; 7 Norwich Medical School, University of East Anglia, Norwich Research Park, Norwich NR4 7UQ, UK; v.vassiliou@uea.ac.uk

**Keywords:** cardiac magnetic resonance, coronary artery disease, chronic coronary syndromes, late gadolinium enhancement, stress cardiac magnetic resonance imaging, diagnosis, prognosis

## Abstract

Cardiac magnetic resonance imaging (CMR) is a useful non-invasive radiation-free imaging modality for the management of patients with coronary artery disease (CAD). CMR cine imaging provides the “gold standard” assessment of ventricular function, late gadolinium enhancement (LGE) provides useful data for the diagnosis and extent of myocardial scar and viability, while stress imaging is an established technique for the detection of myocardial perfusion defects indicating ischemia. Beyond its role in the diagnosis of CAD, CMR allows accurate risk stratification of patients with established CAD. This review aims to summarize the data regarding the role of CMR in the contemporary management of patients with suspected or known coronary artery disease.

## 1. Introduction

**Figure 1:**
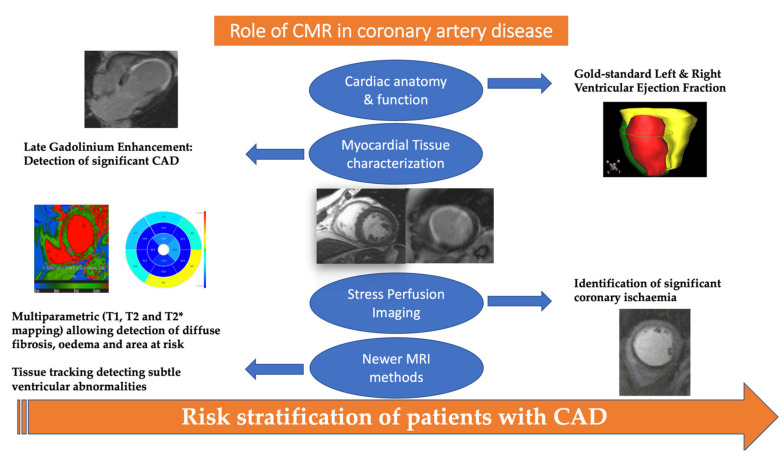
Role of cardiac magnetic resonance in the management of patients with suspected or known coronary artery disease. T2* can be considered an observed T2, whereas the T2 can be considered the natural T2 of the tissue being imaged.

Despite the variability in the prevalence of chronic coronary syndromes in various regions, the disease remains one of the main causes of death worldwide [1]. Invasive coronary angiography is the gold standard in assessing the presence and severity of coronary artery stenoses. However, the invasive nature and potential complications associated with this, as well as the radiation exposure, minimize its role as a first-line screening or diagnostic tool in assessing patients with suspected or known coronary artery disease (CAD). Furthermore, recent data have shown the limited value of concomitant revascularization offered by coronary angiography in chronic coronary syndromes [2]. Computed tomography (CT) angiography is a non-invasive tool for the exclusion of significant coronary stenosis in low-risk patients, but radiation exposure and inadequate imaging in specific populations (irregular rhythm, tachycardia, obesity, calcified coronary arteries) raises major concerns for its widespread use [3]. On the other hand, cardiac magnetic resonance (CMR) imaging is a radiation-free and accurate imaging modality. Among the different CMR modalities, cine cardiac imaging is useful for the assessment of global and regional ventricular function for both the left and the right ventricle, late gadolinium enhancement (LGE) provides useful data for the identification of myocardial scar, the estimation of myocardial infarction size and the assessment of myocardial viability, while stress CMR is an established technique for the detection of perfusion defects indicating myocardial ischemia (Figure 1) [4]. Furthermore, it is well-established that stress CMR can be a cost-effective tool prior to coronary angiography in patients at risk of obstructive CAD [5]. In addition, in patients with suspected CAD, CMR-guided care was found to be superior to National Institute for Health and Care Excellence (NICE) guideline-directed care in reducing unnecessary angiography within 12 months while no significant difference was found compared to myocardial perfusion scintigraphy (MPS)-guided care [6]. According to current guidelines, CMR can also be used for the risk stratification of chronic coronary syndrome patients. More specifically, patients with ≥2 of 16 segments with stress perfusion defects or ≥3 dobutamine-induced dysfunctional segments are classified as having a high event risk and according to the guideline writing committee are likely to benefit from revascularization [3]. This review aims at summarizing the role of CMR in the contemporary management of patients with suspected or known CAD.

## 2. The Role of CMR in the Detection of Significant CAD

The role of CMR as an accurate tool for the evaluation of ventricular dimensions and function has been demonstrated since the 1990s [7,8,9]. High-quality images achieved by CMR can be used for the evaluation of global and regional wall motion abnormalities. CMR imaging is considered as the gold-standard tool for the accurate estimation of left ventricular ejection fraction (LVEF).

In the early 1990s, experimental studies confirmed the role of the gadolinium-enhanced CMR imaging in the identification of infarcted myocardium after coronary occlusion [10]. LGE location can indicate both the presence and extent of myocardial infarct. Furthermore, it is well-established that contrast-enhanced MRI can be used to identify myocardial viability in patients with CAD prior to revascularization procedure [11]. Specifically, it has been found that the percentage of the LV that is both dysfunctional and not hyperenhanced before revascularization is strongly related to the degree of improvement in LVEF following revascularization [11]. However, it should be noted that the absence of LGE rules out myocardial damage relating to previous myocardial infarction (e.g., if a vessel was not fully occluded as seen in NSTEMI, there might be no myocardial scar in that corresponding territory), but cannot be used for ruling out significant CAD, as ischemia itself is not identified using the LGE method.

In this context, perfusion CMR can be used to identify patients with significant, and functional, CAD. A case report published in 1990 highlighted the role of CMR with dipyridamole infusion for the detection of CAD in patients with suspected CAD [12]. Nowadays, perfusion CMR is mainly performed using vasodilators (adenosine or regadenoson) to assess perfusion abnormalities [13,14] or using dobutamine to evaluate regional wall motion abnormalities [15]. Perfusion CMR can discriminate patients with functional coronary stenosis, but if normal, cannot be used to rule out CAD completely as mild non-significant stenosis will still give a normal test. A large prospective real-world study (CE-MARC study) established the superiority of multiparametric CMR consisting of cine imaging, LGE, rest and adenosine stress perfusion, over SPECT as well as its high diagnostic accuracy in CAD [16]. Another multicenter prospective study showed that fewer patients underwent index revascularization in the perfusion CMR-guided group compared to the fractional flow reserve (FFR)-guided group, while perfusion CMR was non-inferior to FFR for the prediction of major adverse cardiac events [17]. A recent Australian study showed that after a positive or inconclusive electrocardiographic stress test in patients with chest pain, stress CMR was the most cost-effective approach for diagnosing significant CAD in Australia’s healthcare system, prior to considering invasive angiography when CMR was positive or inconclusive [18]. A recent meta-analysis concluded stress CMR had a specificity of 91% for ruling out significant CAD with 81% sensitivity [19], whilst the same authors identified coronary CT angiography as sensitive (88%) to detect functionally significant coronary stenosis. Even with the introduction of quantitative myocardial perfusion with CT angiography, the balance is still in favor of CMR; a recent study confirmed that both visual and quantified CMR perfusion outperformed visual CT angiography with perfusion for the diagnosis of hemodynamically significant CAD [20]. Advancements in CMR have also allowed in selected patients to combine coronary anatomy imaging (especially of proximal coronaries) and perfusion CMR to improve detection accuracy of any significant coronary stenosis [21,22,23].

One recent study showed that multiparametric exercise stress CMR accurately correlated with FFR coronary angiography, indicating feasibility [23]. A recent study by Zhang et al. showed that the 3-Tesla (T) CMR coronary angiography improved the sensitivity and diagnostic accuracy for CAD detection compared to myocardial perfusion imaging and LGE alone [24]. Another study which included symptomatic postmenopausal women proposed a combination of two negative stress imaging results (stress CMR with stress echocardiography or single positron emission tomography (SPECT)) for detection of CAD and for risk stratification purposes as this strategy yielded higher accuracy [25]. With regards to perfusion CMR, high-resolution (1.6 × 1.6 mm in-plane) perfusion-CMR has been found to be more accurate as far as the detection of CAD in both single- and multi-vessel disease is concerned, compared to the standard-resolution (2.5 × 2.5 mm in-plane) acquisition [26]. Another recent study compared 1.5T to 3T CMR and revealed that 3T CMR had similar diagnostic performance in detecting significant CAD. However, 3T CMR had a greater performance in patients with multi-vessel CAD without old MI compared to 1.5T CMR [27].

CMR is additionally used for the diagnosis of CAD in specific populations. CMR imaging constitutes an important diagnostic tool in patients with heart failure. Specifically, a recent cohort study showed that in patients with unexplained reduced LVEF, performing coronary angiography only in patients with a presence of myocardial ischemic scar may significantly decrease the number of unnecessary coronary angiographies [28]. Moreover, in patients with decreased LVEF but without LGE, a perfusion CMR or anatomical delineation of coronary arteries is needed. In patients with chronic LV dysfunction due to CAD, delayed enhancement CMR was found to provide the highest sensitivity and negative predicting value for predicting improved segmental LV contractile function after revascularization, whereas low dose dobutamine CMR provides the best specificity and positive predicting value [29]. Stress CMR has been studied for the detection of silent ischemia in patients with antiphospholipid syndrome [30]. In another study which enrolled children with Kawasaki disease and CAD, stress CMR was found to have 100% positive agreement and >90% negative and overall agreement with moderate to severe coronary artery stenoses as depicted by coronary artery angiography [31]. Although further investigation is needed, stress perfusion CMR could possibly be used as an alternative to the coronary angiography follow-up method in patients with left main stenting [32]. The implementation of CMR in evaluating these high-risk patients has the advantage of avoiding radiation and complications related to invasive procedures. However, multicenter prospective studies are needed to validate the utility of CMR in follow-up of patients undergoing PCI of the left main coronary artery. Finally, CMR may play a role in identifying the subgroup of patients with chronic total occlusions (CTO) that may benefit from revascularization [33]. In this setting, the CARISMA-CTO study showed that the implementation of a multi-parameter CMR protocol to study viability/ischemia can help to identify the best candidates for CTO-PCI [34]. This is of great importance considering the high percentage of complications related to complex PCI procedures.

Contrast-enhanced CMR can also be used for the identification of microvascular dysfunction following reperfusion while microvascular obstruction was found to predict major adverse cardiovascular events and cardiac death [35]. In women with ischemia and non-obstructive CAD, CMR-derived circumferential strain has been found to predict coronary microvascular dysfunction [36]. Furthermore, patients with typical angina and risk factors for microvascular disease were found to have reduced stress myocardial perfusion and myocardial perfusion reserve compared to healthy controls [37] and this perfusion defect is in a characteristic global pattern. Therefore, in the last 30 years, the update of CMR has increased significantly. Newer developments have enabled accurate visualization of the important proximal and mid parts of the coronaries and quantified perfusion provides incremental value in addition to the established anatomical, volumetric analysis and LGE for scar identification and viability.

CMR is a key diagnostic tool in the evaluation of patients presenting with myocardial infarction with non-obstructed coronary arteries (MINOCA) because it not only provides useful data about the potential causes but may also provide confirmation of the diagnosis of AMI [38]. It has been found that CMR performed within seven days of presentation can contribute to a diagnosis in nearly 90% of patients presenting with acute chest pain, elevated serum troponin and non-obstructed coronary arteries [39]. Interestingly, the role of CMR in this setting was confirmed by the results of a meta-analysis [40]. Specifically, this study showed that CMR findings reasserted myocarditis as the leading diagnosis in patients with MINOCA [40].

## 3. Role of CMR in the Risk Stratification of Patients with CAD

Myocardial fibrosis assessment can improve the prognostic role of CMR in patients with CAD. Catalano et al. suggested that beyond clinical and echocardiographic assessment, LGE could be used to assess myocardial viability and to further stratify the risk of death in patients with stable CAD [41]. Stress CMR can be used for risk stratification purposes as well. Moderate to severe perfusion defects in stress CMR has been found to predict cardiovascular events [42,43]. In addition, characterization of healthy non-infarcted myocardium by native T1 imaging can be potentially used as a predictor of outcome in patients with CAD, beyond the traditional risk tools [44]. CMR-derived coronary flow reserve is another independent predictor of major adverse cardiovascular events in patients with known or suspected CAD [45]. Moreover, the predictive values of coronary flow reserve (as assessed by CMR) and stress perfusion CMR for major adverse cardiovascular events were comparable in patients with known CAD [46]. Adding aortic stiffness (as expressed by pulse wave velocity) to stress CMR could improve prediction of mortality, acute coronary syndrome, heart failure, need for coronary revascularization and stroke [47]. Furthermore, it has been suggested that the implementation of a score combining clinical (age, sex, diabetes mellitus and left ventricular ejection fraction) and stress CMR data (ischemic burden defined as the number of segments with stress-induced perfusion defects) could predict the risk of long-term all-cause mortality in patients with known or suspected CAD [48]. Appreciating that adenosine can be contraindicated in some individuals, newer pharmacological vasodilators like regadenoson stress CMR can also be used to predict adverse events in patients with known or suspected CAD. Specifically, normal perfusion can identify patients with low risk of coronary revascularization, non-fatal myocardial infarction, and cardiovascular death [49]. On the other hand, dobutamine CMR (nowadays used predominantly if there are contraindications for adenosine or renal failure prohibiting the use of gadolinium) plays an important role in predicting cardiac death and nonfatal myocardial infarction during long-term follow-up [50]. A South Asian study showed that the implementation of CMR-derived factors such as infarct size and wall motion score index with LVEF can improve the risk stratification of patients with CAD and can lead to cost-effective therapeutic strategies [51].

In patients with ischemic symptoms but no significant CAD, stress perfusion CMR was found to be an independent predictor of major adverse cardiovascular events [52]. Furthermore, the role of dobutamine CMR can also be utilized to assess the functional significance of anomalous coronaries, although physiological exercise CMR if available is the preferred modality. The safety and prognostic value of stress CMR regarding the occurrence of major adverse cardiovascular events in heart failure with reduced ejection fraction (HFrEF) have also been studied [53]. Even in the elderly, dipyridamole stress perfusion CMR was characterized as a safe diagnostic tool that could additionally identify patients with a lower event rate of future cardiovascular event or death [54]. A meta-analysis showed that a negative stress CMR study was associated with very low risk of cardiovascular death and myocardial infarction in patients with known or suspected CAD [55]. Similarly, a meta-analysis performed by Gargiulo et al. showed that stress CMR has a high negative predictive value for adverse cardiac events, and the absence of inducible perfusion defect or wall motion abnormality shows a similar ability to identify low-risk patients with known or suspected CAD [56].

A multi-center retrospective study concluded that a stress CMR without evidence of ischemia or LGE identified patients at low risk of adverse cardiac events [57]. Similarly, a meta-analysis showed that the presence and size of LGE in patients with CAD can predict mortality and major adverse cardiovascular events [58]. It has been found that without revascularization, the presence of dysfunctional viable myocardium as recognized by delayed enhanced CMR was an independent predictor of mortality in patients with ischemic left ventricular dysfunction [59]. The number of scar segments as identified by LGE has been found to be an independent predictor of cardiovascular events after CABG in patients with a history of myocardial infarction [60]. Finally, CMR can also be used to identify patients at risk of arrhythmias; it has been found that in CAD patients, scar extent studied by LGE was significantly associated with appropriate implantable cardioverter defibrillator (ICD) therapy and as a result, it can be used for identifying patients at high risk of future lethal arrhythmic events [61,62,63,64]. Specifically, Alexandre J. et al. showed that in patients with CAD and an ICD implanted either for primary or for secondary prevention of sudden cardiac death (SCD), scar extent studied by LGE-CMR was significantly associated with appropriate ICD therapies [61]. Similarly, another retrospective study showed that both the percent of scar and number of transmural scar segments were significantly associated with the occurrence of appropriate ICD therapies [62]. In the setting of ischemic cardiomyopathy with reduced ejection fraction, myocardial scar burden may predict gender-based differences in survival benefit from ICDs [64].

Furthermore, quantification of the peri-infarct zone has also been found to predict appropriate ICD therapy in ischemic cardiomyopathy [63]. These findings suggest the use of LGE as a tool for the identification of CAD patients who are at high risk of fatal arrhythmias. However, more studies are needed to confirm the role of LGE for improving ICD treatment decisions.

## 4. Limitations of CMR Imaging

Advantages and disadvantages of the commonly used imaging modalities in the assessment of CAD patients are provided in Table 1. Limited access or availability and the high cost are important barriers to CMR widespread use. Whilst CMR can provide accurate answers to multiple cardiac questions, the magnetic nature means that it might not be suitable, or might indeed be contraindicated, for some patients. Specifically, absolute contraindications include: no MRI-compatible implantable electronic devices (although still some centers are able to scan non-compatible devices following careful assessment and modifications to the magnet sequences), metallic intraocular foreign bodies, implantable neurostimulation systems, cochlear implants/ear implant, drug infusion pumps, catheters with metallic components (Swan-Ganz catheter), metallic fragments such as bullets, cerebral artery aneurysm clips, magnetic dental implants, tissue expander, artificial limb, and piercing [65]. Relative contraindications include: newly implanted coronary and peripheral artery stents, programmable shunts, airway stents or tracheostomy (except plastic), intrauterine device, ocular prosthesis, surgical clips or wire sutures, joint replacement or prosthesis, inferior vena cava filter, tattoos, colonoscopy procedure in the last eight weeks, and claustrophobia [65]. In the case of gadolinium infusion, the following patients should be carefully assessed or deferred: patients on dialysis, patients with a history of renal disease or an estimated glomerular filtration rate below 30 mL/min/1.73 m^2^, patients who had received a dose of contrast in the last 24 h, patients who had a previous allergic or anaphylactic reaction to gadolinium, patients who have risk factors for nephrogenic systemic fibrosis (NSF), patients with diabetes mellitus or hypertension who are receiving treatment with medications, and patients who are pregnant [65].

## 5. Recent Advances in CMR Imaging

Although the volumetric evaluation of LVEF is the cornerstone for assessing the cardiac function, it is recognized that additional information, over and above ejection fraction, can be beneficial. In this context, strain imaging can provide useful data for regional abnormalities as well as to identify ventricular dysfunction in a pre-clinical stage [66,67]. However, normal values for the method and software cutoff need to be established. On the other hand, quantitative myocardial perfusion is an accurate technique for the diagnosis of occlusive CAD while it can also be used for ruling out significant CAD [68]. Artificial intelligence has been implemented in perfusion CMR mapping [69] and provides instantaneous quantification of myocardial perfusion by CMR. It has been found that in patients with known or suspected CAD, quantitative myocardial blood flow measurement using artificial intelligence is an independent predictor of adverse cardiovascular outcomes [69]. Coronary imaging by CMR has also been improved in the last 10 years and it is likely that in the next decade, the quality and speed by which this can be obtained will also be improve, making it more clinically available. In the longer term, faster CMR protocols which will involve less breath-holding will be facilitated, making a CMR study cheaper, quicker (and thus available to further people) and more accurate than what it is currently [22].

## 6. Conclusions

CMR is an accurate and cost-effective imaging modality that could be used to identify patients who will benefit from an invasive coronary angiography. Furthermore, it can be used for risk stratification purposes in patients with known CAD. Recent advances have increased the utility of CMR imaging in clinical practice in patients with suspected or known CAD, and significant improvements on the horizon making CMR quicker and cheaper signify that its role in clinical practice will only continue to expand.

## Figures and Tables

**Table 1:** Advantages and disadvantages of the commonly used imaging modalities in the management of coronary artery disease patients.

	Advantages	Disadvantages
Stress echocardiography	Wide access No radiation exposure Low cost	Poor ultrasound windows in specific conditions (obesity, COPD, etc.) Dependent on operator skills Limited tissue characterization
SPECT	Wide access Standardized protocols Large evidence base for diagnostic accuracy and risk stratification	Radiation Suboptimal identification of multi-vessel coronary disease
CMR	Both anatomic and functional information Highly reproducible Superior tissue characterization No ionizing radiation	High cost Acquisition time Use of contrast Difficulties mainly in arrhythmias and obesity Contraindications (vascular clips, implants, etc.)
CCTA	High negative predictive value in patients with low pre-test probability	Radiation Limited availability Difficulties mainly in arrhythmias (tachycardia) Assessment limited in cases with extensive coronary calcification

SPECT, Single-Photon Emission Computed Tomography; CMR, Cardiac Magnetic Resonance; CCTA, Coronary Computed Tomography Angiography.

## Data Availability

This study did not report any data.

## Abbreviations

SPECT, single-photon emission computed tomography; CMR, cardiac magnetic resonance; CCTA, coronary computed tomography angiography; COPD, chronic obstructive pulmonary disease
